# Acute Limb Ischemia in COVID-19 Patients: A Single University Center Experience

**DOI:** 10.7759/cureus.32829

**Published:** 2022-12-22

**Authors:** Hamza Naouli, Hamid Jiber, Abdellatif Bouarhroum

**Affiliations:** 1 Vascular Surgery, Sidi Mohamed Ben Abdellah University Faculty of Medicine and Pharmacy of Fez, University Hospital Center Hassan II of Fez, Fez, MAR

**Keywords:** surgical embolectomy, arterial thromboembolism, covid 19 - acute limb ischemia, covid coagulopathy, covid-19

## Abstract

Introduction

Severe acute respiratory syndrome coronavirus 2 (SARS-CoV-2) infection is currently known to lead to high rates of thrombotic complications. Of those, acute limb ischemia (ALI) was most frequently reported. Several case reports or case series had already described high mortality and amputation rates. The purpose of our study was to highlight the epidemiological, clinical, and management characteristics of coronavirus disease 2019 (COVID-19)-related ALI patients.

Methods

This was a monocentric, observational, and retrospective study. Records of all patients ≥18 years of age admitted with ALI and a confirmed diagnosis of COVID-19 infection between March 2020 and December 2021 were retrospectively examined. Data collected included demographics, co-morbidities, biological findings, COVID-19 pneumonia and ALI severity, anatomical location of arterial thromboembolism, treatments, and outcomes.

Results

During the study period, 22 patients with ALI infected with COVID-19 were evaluated. The median age was 67 years (45-88) and 18 (81.8%) were men. The main comorbidities were diabetes mellitus (36.4%), smoking (22.7%), and arterial hypertension (18.2%). All 22 patients were already diagnosed positive for SARS-CoV-2. The median duration between COVID-19 diagnosis and ALI symptom onset was six days (1-13 days). The computed tomography (CT) extent of pulmonary lesions was assessed according to the French Society of Chest Imaging. The ischemic syndrome was classified on Rutherford Stage IIA (30.4%) and IIB (43.5%). Regarding thrombotic locations, ALI had occurred essentially in the lower limbs (95% vs. 5%). A revascularization procedure was performed in 14 patients (63.6%) of the patients, and primary amputation was unavoidable in five patients (22.7%). Three patients (13.6%) did not undergo operative management, two because of their hemodynamic instability and one rejected surgery. We performed 23 revascularization procedures for 14 patients and three primary amputations. Thromboembolectomy (TE) was the technique of choice (92.8%). Below-the-knee (BTK) femoropopliteal bypass was performed in one patient. Selective tibial vessel thrombectomy was performed in four patients (28.6%). The mortality rate was 27.3%. Among survivors, two secondary amputations were needed with a limb salvage rate of 68.2%.

Conclusion

By the apparent end of the pandemic, our study further supports the increased risk of ALI in COVID-19-positive patients. Moreover, the results affirm the unfavorable outcomes highly impacted by rethrombosis, reinterventions, and consequently high rates of amputations and mortality.

## Introduction

Despite the sharp decrease in the cases number, coronavirus disease 2019 (COVID-19) is still considered a pandemic by WHO since March 2020 [1]. The disease caused by severe acute respiratory syndrome coronavirus 2 (SARS-CoV-2) is a multisystemic disorder primarily involving the respiratory, hematologic, and cardiovascular systems [2]. Therefore, multiple extra-respiratory involvements induced by the hypercoagulable state at the cardiovascular system level affect about 49% of patients and worsen the prognosis [3-4]. Vascular surgeons worldwide were involved in the treatment of acute limb ischemia (ALI) as a complication of SARS-CoV-2, and several studies had reported a poor prognosis and high rates of major amputations and mortality. The purpose of this paper was to report our experience in the management of COVID-19-related ALI.

## Materials and methods

Since the first published cases, our hospital has implemented a standardized evaluation protocol for all patients admitted with ALI. This program included, besides CT angiography of the abdominal aorta and lower limbs, a chest CT and reverse transcription-polymerase chain reaction (RT-PCR). After this preoperative evaluation, all patients who required urgent revascularization were discussed in a multidisciplinary team (vascular surgeon, anesthesiologist, and intensive care unit practitioner). Moreover, in view of the risk incurred, informed consent was obtained from all patients considered for surgery.

This was a single-center, observational, and retrospective study. Records of all patients ≥18 years of age admitted with ALI and a confirmed diagnosis of COVID-19 infection between March 2020 and December 2021 were retrospectively examined. Data collected included demographics, co-morbidities, biological findings, COVID-19 pneumonia and ALI severity, anatomical location of arterial thromboembolism, treatments, and outcomes.

Surgical techniques included thrombo-embolectomy, bypass, and amputations. Procedures were performed under general and loco-regional anesthesia. The femoral and BTK popliteal approaches were the most used techniques. The medical devices used were exclusively embolectomy catheters, and a peripheral bypass was performed with the great saphenous vein. Revascularization success criteria were assessed clinically by physical examination.

All collected data were entered into a spreadsheet (Microsoft Excel 2013, Microsoft Corporation, Redmond, WA), and statistics were performed as a descriptive analysis using SPSS (IBM Corp, Armonk, NY). Continuous variables were expressed as medians and interquartile ranges. Categorical variables were summarized as counts and percentages.

## Results

**Figure 1:**
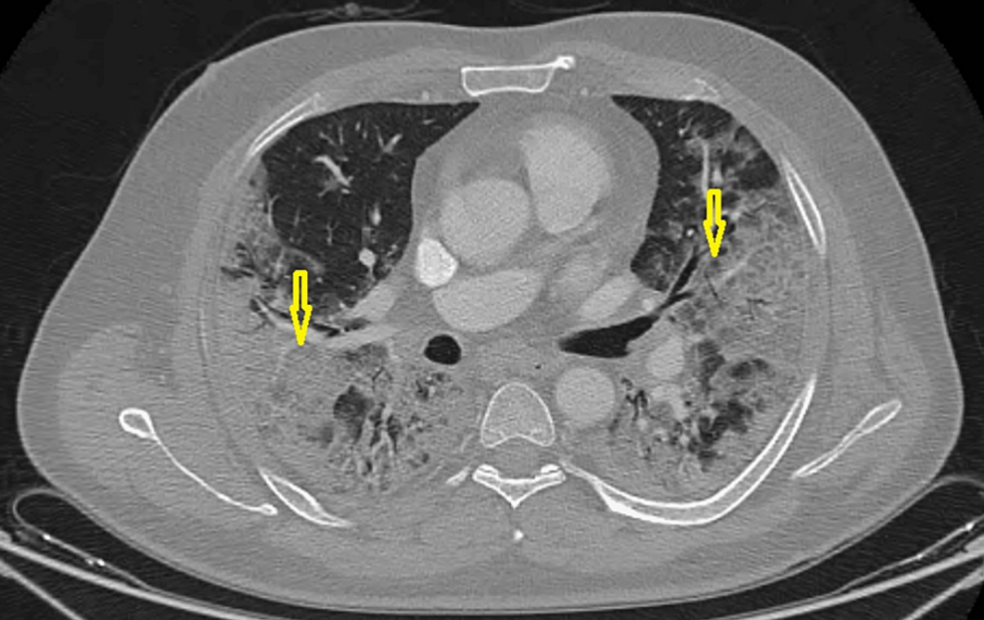
Preoperative chest computed tomography on transverse thin-section scans showing extensive ground-glass opacities of both lungs (yellow arrows)

In the study period, 22 consecutive patients who tested positive for SARS-CoV-2 were referred to our department for the management of ALI. Of those, 18 (81.8%) were men with a median age of 67 (45-88). The main comorbidities were diabetes mellitus (2; 36.8%), smoking (22.7%), and arterial hypertension (18.1%). Besides this, a history of hyperthyroidism, subarachnoid hemorrhage, and chronic obstructive pulmonary disease was found in one patient each. Until the end of the study, all the patients were not vaccinated yet against SARA-CoV- 2. ALI revealed COVID-19 pneumonia in four (18.2%) patients, however, 18 (81.8%) developed ischemia during hospitalization after an average of six days. The diagnosis of COVID-19 infection was made through RT-PCR in all patients. The laboratory test findings are shown in Table 1. The CT extent of pulmonary lesions was assessed according to the French Society of Chest Imaging. Therefore, the pneumonia was classified as extensive (26-50%) in 45.5%, severe (51-75%) in 31.8%, moderate (1-25%) in 18.2%, and critical (˃75%) in 4.5% of patients (Figure 1).

**Table 1:** Demographic data, comorbidities, risk factors, clinical characteristics, surgical management, and outcomes CV: Cardiovascular; HBP: High blood pressure; SAH: Subarachnoid hemorrhage; COPD: Chronic obstructive pulmonary disease; CVE: Cardiovascular events; ALI: Acute limb ischemia; OADD: Oral antidiabetic drugs; ULI: Upper limb ischemia; LLI: Lower limb ischemia; AFT: Aortic floating thrombus; Pop: Popliteal; TA: Tibial arteries; SCA: Subclavian artery; Fem: Femoral; Bif: Bifurcation; TE: Thromboembolectomy; TF: Transfemoral; TT: Transtibial; TM: Transmetatarsal; ICU: Intensive care unit

	N (%)
Mean age (years)	66,95
Sex	
Male (%)	18 (81.8%)
Female (%)	4 (18.2%)
CV risk factors	
HBP	4 (18.2%)
Diabetes	8 (36.4%)
Smoking	5 (22.7%)
Medical History	
Hyperthyroidism	1
SAH	1
COPD	1
Previous CVE	0
Previous medication	
OADD/Insulin	4/4
Antihypertensive therapy	4
Covid19 severity degree (%)	
Moderate 10-25%	4 (18.2%)
Extensive 26-50%	10 (45.5%)
Severe 51-75%	7 (31.8%)
Critical >75%	1 (4.5%)
Days (disease onset-ALI)	6
Anticoagulation before ALI	
Prophylactic	14 (63.6%)
Therapeutic	4 (18.2%)
None	4 (18.2%)
Clinical Event	
ULI	1
LLI	20
ULI + LLI	1
Rutherford Staging	
I	2 (8.7%)
IIa	7 (30.4%)
IIb	10 (43.5%)
III	4 (17.4%)
Anatomic location	
Aorta (AFT)	4 (18.2%)
Ilio-femoral	7 (31.8%)
Pop	13 (59.1%)
TA	8 (36.4%)
SCA	2 (9.1%)
BA/RA/UA	1 (4.5%)
Cases with multiple embolic locations (%)	14 (63.6%)
Laboratory findings event	
White-cell count (/mL)	16415.79 (7500-29000)
D-dimer (ng/mL)	2268.11 (8.67-4500)
C-reactive protein (mg/L)	179.57 (51-419)
Serum ferritin (ng/mL)	1173.55 (621-1971)
Non-interventional therapy	3
Revascularization approach	23 procedures
Fem bif TE	9 (39.2%)
Pop and TA TE	11 (47.8%)
BTK FP Bypass	1 (4.3%)
Brachial TE	2 (8.7)
Fasciotomy	6 (42.8%)
Amputation	7 (31.8%)
Primary	5 (22.7%)
Secondary	2 (9.1%)
TF	3
TT	3
TM	1
ICU admission (%)	12 (54.5%)
Outcomes	
Hematoma	1
Amputation stump necrosis/infection	3
Death (%)	6 (27.3%)

**Figure 2:**
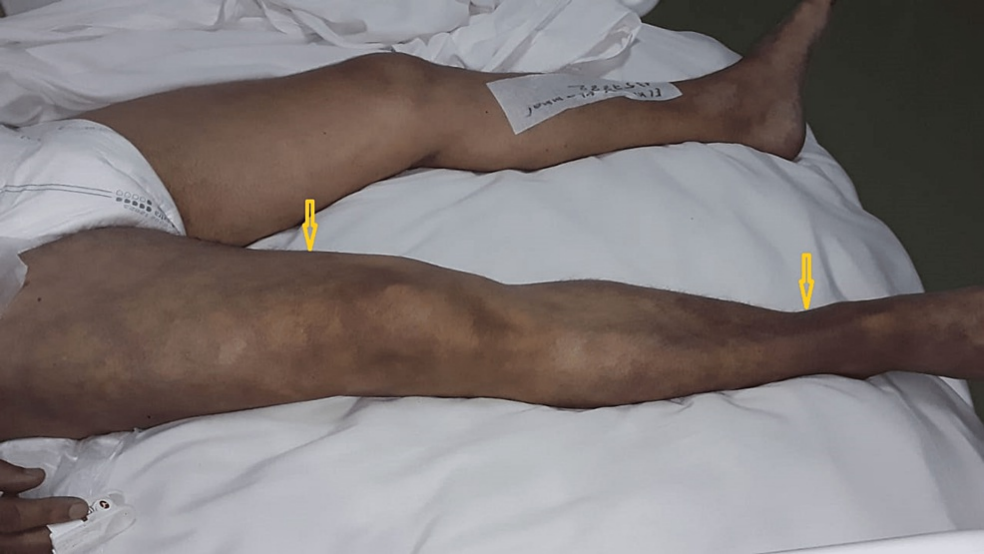
Clinical appearance of severe acute limb ischemia in a hospitalized patient with COVID-19 Yellow arrows: cyanosis

Of all patients, intensive care unit (ICU) preoperative admission was necessary for eight (36.4%). Postoperatively, ICU transfer was needed for four cases (18.2%). Before the ischemic event, 14 patients (63.6%) received prophylactic doses of low molecular weight heparin (LMWH), however, therapeutic doses were proposed for four cases (18.2%). Nevertheless, four patients (18.2%) did not have any heparin protocol. All patients were evaluated according to the Rutherford classification; it was found that the majority of them were classified as Stages IIA (30%) and IIB (42.8%) (Figure 2).

**Figure 3:**
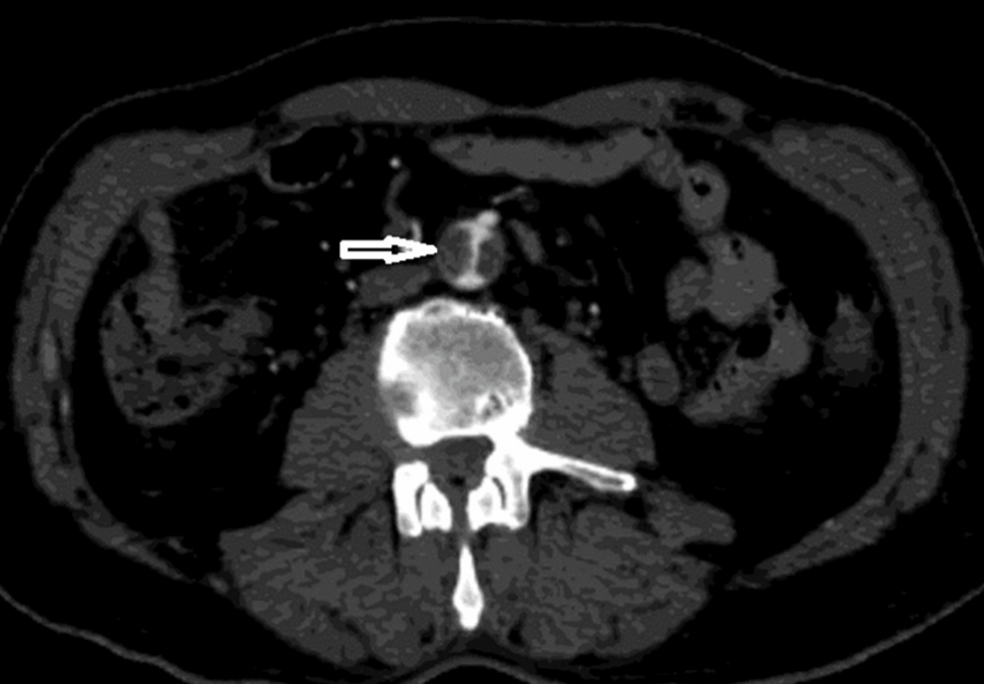
CT angiography on axial view revealing an intraluminal abdominal aorta thrombus (white arrow)

**Figure 4:**
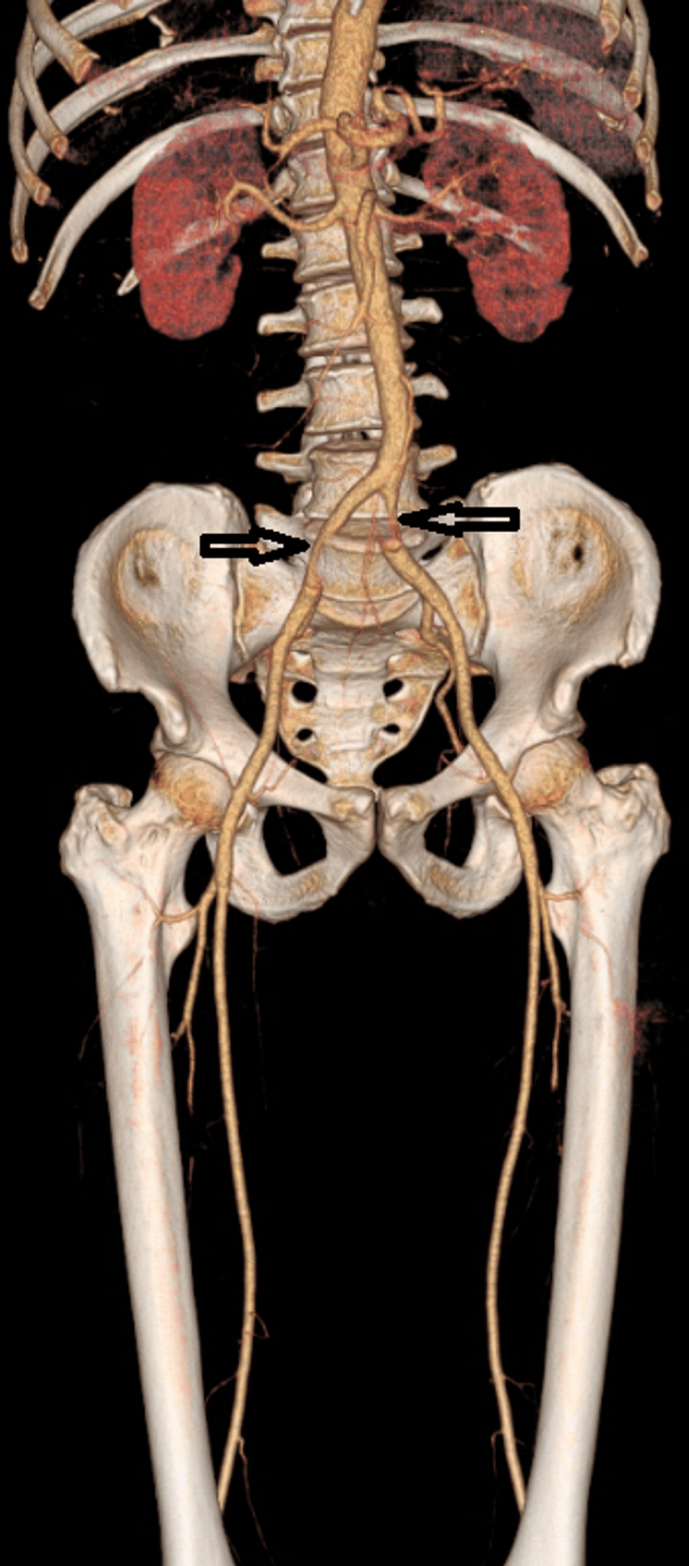
CT angiography on 3D reconstruction showing a bilateral common iliac artery partial thrombosis (black arrows)

**Figure 5:**
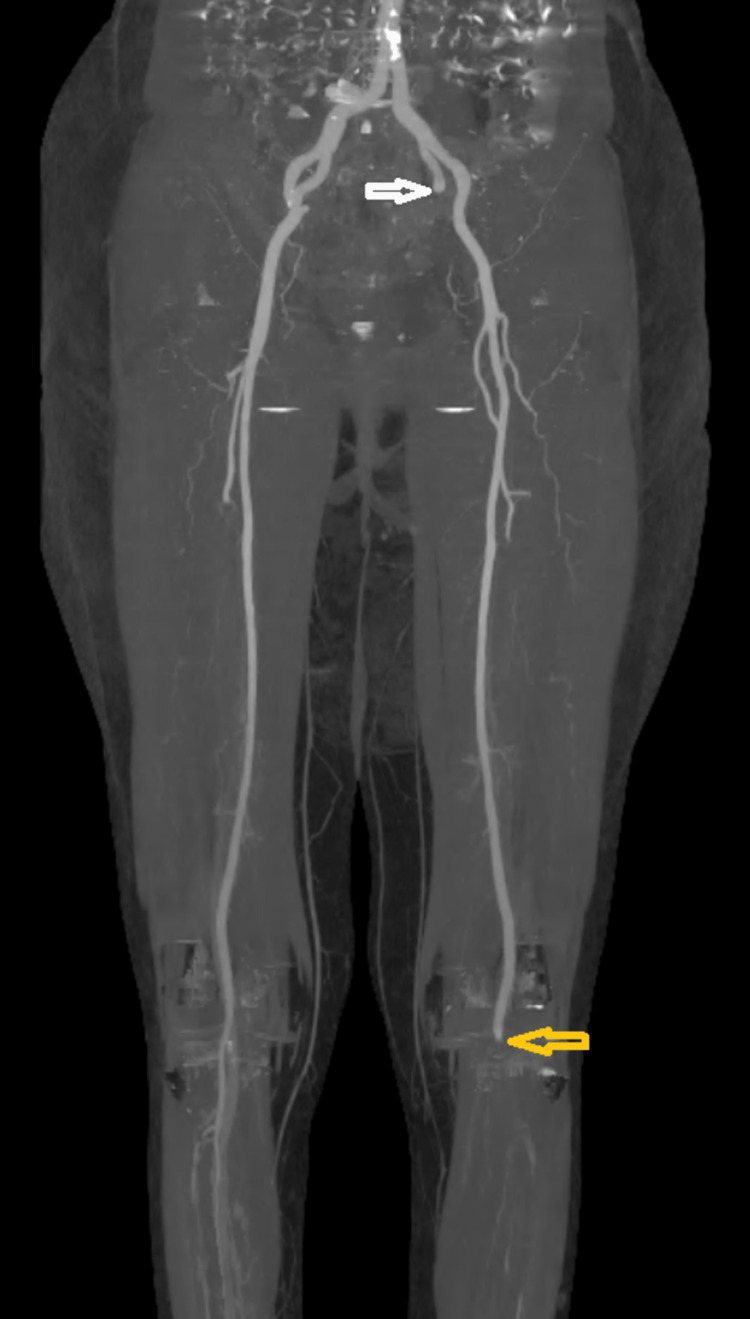
3D CT angiography revealing a left hypogastric (white arrow) and popliteal (yellow arrow) artery occlusion

The lower limbs were the most affected (95.4%). In our cohort, preoperative CT angiography was the golden standard, which was performed for 19 patients (86.4%). However, two patients underwent a Doppler ultrasound. The remaining patient was too unstable for transport to imaging, and the diagnosis was based on clinical signs of acute ischemia. Over two-thirds (63.6%) of patients had more than one segment thrombosis, and ischemia had occurred simultaneously in the upper and lower extremity in one case. The most involved vessels were the popliteal artery (59.1%), iliofemoral segment (31.8%), and tibial arteries (36.4%). Moreover, abdominal aortic floating thrombus was found in four cases (18.2%) (Figures 3-5).

Concerning upper extremity ischemia, thrombosis was located in the subclavian artery in one patient. However, in the second case, besides SCA occlusion, thrombosis also involved brachial and forearm arteries. We have performed 23 revascularization procedures for 14 patients. Thromboembolectomy (TE) was the technique of choice (92.8%), essentially through the medial below-the-knee popliteal approach in 47.8% of cases and by femoral bifurcation exposure for 39.2% of patients. A below-the-knee femoropopliteal bypass was performed in one patient. Selective tibial vessel TE was performed in four patients (28.6%). Fasciotomy was performed in six (42.8%) cases. Upper limb ischemia was treated through the direct open brachial artery approach. After limb reperfusion, systemic anticoagulation was performed with unfractionated heparin (UFH) infusion (n=10) and with LMWH in four patients. Three patients (13.6%) did not undergo operative management. Of those, two were because of their hemodynamic instability and the third one rejected surgery. There were seven amputations (31.8%), five of them were primary and two were secondary (Table 2).

**Table 2:** Primary and secondary amputation among patients with COVID-19-related ischemia Pop: Popliteal; TA: Tibial arteries; Fem: Femoral; Bif: Bifurcation; TE: Thromboembolectomy; TFA: Transfemoral amputation; TTA: Transtibial amputation; SFA: Superficial femoral artery; CFA: Common femoral artery; ALI: Acute limb ischemia; ICU: Intensive care unit

	Amputation						
	Primary					Secondary	
	Case N 5	Case N 7	Case N 15	Case 17	Case 18	Case N 8	Case N 19
Age	80	71	60	76	75	58	69
Covid19 severity	70 %	10% then 85%	50%	25-50%	10-25%	75%	25%
ICU admission	Yes	Yes	No	No	No	Yes	Yes
Thrombosis location	Pop - TA	CFA - SFA	Pop-TA	CFA	TA	Pop - TA	Pop - TA
Rutherford stage	III	III	III	IIb	IIb	IIb	IIb
Revascularization	None	None	None	Fem bif TE	None	Pop – TA TE	Pop – TA TE
Amputation	TFA	TFA	TFA	TTA	TTA	TTA	TFA
Indication	Irreversible ALI	Irreversible ALI	Irreversible ALI	Re-occlusion	Severe ALI	Reperfusion syndrome	Reperfusion syndrome
Outcome	Death	Death	Alive	Alive	Alive	Alive	Death

The rate of limb salvage was 68.2%, and overall in-hospital mortality was 27.3%. Additionally, deaths were related to acute respiratory distress syndrome in four cases and secondary to reperfusion injury in two patients. At one-month follow-up, re-intervention was required for four patients. Of those, three amputations of stump necrosis were treated by surgical debridement, and one operative site hematoma was evacuated. It’s noteworthy that in the same period, non-COVID-19 ALI was managed. They were 61 patients (upper: 13; Lower: 48). Of those, the major amputation rate was 18% and mortality was estimated at 11.5%.

## Discussion

Since the onset of the COVID-19 pandemic, multiple reports have circulated demonstrating increased rates of thromboembolic events in patients with COVID-19 [5-6]. The cumulative incidence of thrombotic complications in critically ill patients with COVID-19 was 31% despite systemic thromboprophylaxis, including 27% venous thromboembolism and 4% arterial thrombotic events [7]. In fact, during the pandemic, ALI occurred approximately five times more frequently in COVID-positive patients [8].

The exact mechanism of acute ALI in patients with COVID-19 infection is not yet well-understood, but several theories have been proposed [9]. Thus, a cytokine storm induced by activated macrophages in a systemic inflammatory state may be the most credible hypothesis [10]. Moreover, viral invasion of endothelial cells, endothelial injury from inflammation, and free-floating aortic thrombus were also raised [11-12]. Consequently, COVID-19 patients have more likely a pro-coagulant state, which leads to arterial thrombosis [13].

Regarding patient predisposition, one of the main findings reported by Attisani et al. is that the majority of patients involved did not suffer from any of the classical risk factors for ALI; some of them were either on prophylactic anticoagulation or antiplatelet therapy at baseline [14]. Hence, several papers reported few risk factors in their cohort. In our study, old age, male sex, and high blood pressure were the most encountered cardiovascular risk factors. These findings were also reported by other authors [15-17]. In our study, ALI occurred predominantly in an elderly population. Moreover, diabetes (36.4%), smoking (22.7%), and high blood pressure (18.2%) were the most frequent cardiovascular risk. Although 22.7% of patients had no medical story, male sex predisposition was also mentioned in COVID-19-related ischemia. Therefore, there was a clear male predominance in most of the studies [15,18-19]. Similarly, 81.8% of patients in our study were men.

As shown in the Attisani et al. review, there is no currently available information about the temporal correlation between SARS-CoV-2 and the boost of ALI; however, the majority of patients in this review (64%) developed ALI during the acute phase of SARS-CoV-2 [14]. On the other hand, some published reports documented delayed ALI in patients recovered from SARS-CoV-2 with a negative nasopharyngeal swab suggesting the fact that the pro-inflammatory state could persist even weeks after infection [20,21].

ALI severity was assessed in some studies using the Rutherford classification. Thus, most patients were classified as Rutherford IIA (28% to 77%) and IIB (17% to 75%) [8-18,22]. Compared to these series, the ALI degree of our patients was classified as Stage IIA (30%) and IIB (42.8%).

Regarding anatomic distribution, ALI was located essentially in the lower extremities [23-24]. In such localization, thrombotic events tend to be more extensive and with a greater clot burden [25]. In addition, the femoropopliteal trunk was reported to be the most involved segment [22]. In one study, the arterial occlusion affected this region in approximately two-thirds of patients, followed by below-the-knee arteries in 29.4% of cases and “desert foot” in 23.5% of patients [8]. We documented similar results in our cohort. Therefore, the most involved segment was the popliteal artery (59.1%), followed by tibial arteries in 36.4% of cases.

The results of our observational study support other cohort findings in terms of clinical presentation, anatomic distribution, high rates of re-thrombosis and major amputations, and finally poor vital prognosis (Table 3).

**Table 3:** Comparison of clinical presentation, ischemia severity and distribution, type of revascularization procedures, and outcomes with some of the largest cohorts in the literature NR: Not reported; LL: Lower limb; UL: Upper limb; POPA: Popliteal artery; BA: Brachial artery; UA: Ulnar artery; RA: Radial artery; AFT: Aortic floating thrombus; TA: Tibial arteries; SCA: Subclavian artery; BTK: Below the knee; FP: Femoropopliteal; TE: Thromboembolectomy; FE: Femoral endarterectomy; IST: In situ thrombolysis.

Study/year	Country	No. Of ALI patients	Mean age (years)	Rutherford stage (%)	Limb affected (LL/UL)	Arteries involved (n/%)	Revascularization procedure (n)	Amputation (%)	Death (%)
Bellosta et al. (2020) [8]	Italy	20	75	IIa (10%), IIb (75%), III (15%)	LL and UL (NR)	Infrainguinal (13), Aortoiliac (3), TA(5)	TE (15), FP bypass (2), IST (4), Kissing stents (2), FE (1), BTK angioplasty (1)	5.8%	40 %
Sanchez et al. (2020) [18]	Peru	30	60	IIa (44.4%), IIb (33.3%), III (22.3%)	22/8	Most affected: POPA (10.7%), BA (8.7%)	TE (23)	30%	23.3%
Etkin et al. (2020) [19]	USA	42	NR	NR	35/7	Aortoiliac (8), Femoral (12), POPA (15), Above elbow (4), UA and RA (3)	TE (9), IST (2)	18%	50%
Kahlberg et al. (2021) [26]	Italy	41	NR	NR	LL and UL (NR)	NR	NR	NR	29.3%
Goldman et al (2020) [30]	USA	16	70	NR	16/0	From the POPA and proximally (15), Distally from the POPA (1)	TE (6)	25%	38%
Our study	Morocco	22	67	I (8.7%) IIa (30.4%), IIb (43.5%), III (17.4%)	21/2 (1 Simultaneous LL and UL ischemia )	AFT (4), POPA (13), TA (8), Iliac (7), Femoral (7), SCA (2), BA-UA-RA (1)	TE (13), BTK FP bypass (1)	31.8%	27.3%

ALI associated with COVID-19 pneumonia is a critical situation. One of the problematic decisions was the choice of intervention. This last was often directed by three requirements, which were limiting the number of stressful procedures and the exposure of medical personnel with reasonable resource use [26]. On the other hand, their management is heterogeneous and depends mostly on the patient’s overall stability, degree of ischemia, and limb viability. Indication of surgical intervention has to consider the severity of the systemic illness of the patient [14]. Concerning the non-interventional option, Galyfos et al. have shown a worse prognosis in terms of mortality and amputation risk [27].

The majority of patients were submitted to surgical revascularization, TE was the preferred technique (31%) [14]. In this sense, surgical thrombectomy alone had been used in 76% of cases by one vascular surgery team. Moreover, in their study, fasciotomy was required in 20% of patients [18-19]. Similarly, we have performed TE exclusively without adjunctive procedures in 92.8% of revascularized patients. Although, some authors have demonstrated the importance of hybrid therapies that associate open surgery with angioplasty or stenting in selected significant lesions [19,22], Attisani et al. reported that completion angiogram usually showed a “desert foot” condition caused by the absence of microcirculation [8]. These findings forced vascular surgeons worldwide to change their therapeutic approach to ALI in SARS-CoV-2 [14]. Some authors reported routine application of locoregional thrombolysis in case of failure of the primary intervention to achieve satisfying results [8-28].

In our cohort, between primary and secondary amputations, the amputation rate was 31.8% (n = 7). Other authors have also reported this high amputation rate. Galyfos et al., in their cohort, have encountered a 23.5% amputation rate [27]. This was related to the low successful revascularization rate in COVID -19 patients reported by many authors [29]. Besides, the mortality rate in our cohort was 27.3%. This result follows findings reported by other case series that described mortality rates between 18.8% and 40% [17,30].

This cohort has several limitations. The main one is the study design; it is observational descriptive and retrospective review without a control group. Therefore, statistically significant conclusions about this disease complication and its outcomes cannot be made.

## Conclusions

COVID-19-positive patients are at increased risk of ALI and resultant mortality, given the hypercoagulable state. The lower extremities were the most involved territory. Revascularization techniques included open TE, adjunctive endovascular therapy, and in some cases, thrombolysis. Despite this therapeutic panel, several studies had reported a poor prognosis and high rates of major amputations. Regarding the high rate of “desert foot” condition caused by the absence of microcirculation, loco-regional thrombolysis seems to be an interesting alternative. Mortality rates were related to both pulmonary involvement severity and ALI with its systemic consequences.
